# Improvement of wound healing by capsaicin through suppression of the inflammatory response and amelioration of the repair process

**DOI:** 10.3892/mmr.2023.13042

**Published:** 2023-06-30

**Authors:** Chi-Jung Huang, Chi-Ming Pu, Su-Yi Su, Shih-Lun Lo, Cheng Hung Lee, Yu-Hsiu Yen

**Affiliations:** 1 Department of Medical Research, Cathay General Hospital, Taipei 10630, Taiwan, R.O.C.; 2 Department of Biochemistry, National Defense Medical Center, Taipei 11490, Taiwan, R.O.C.; 3 Division of Plastic Surgery, Department of Surgery, Cathay General Hospital, Taipei 10630, Taiwan, R.O.C.; 4 School of Medicine, College of Life Science, National Tsing Hua University, Hsinchu 30013, Taiwan, R.O.C.; 5 Department of Anatomy and Cell Biology, College of Medicine, National Taiwan University, Taipei 10617, Taiwan, R.O.C.; 6 School of Medicine, College of Medicine, Fu Jen Catholic University, New Taipei City 24205, Taiwan, R.O.C.

**Keywords:** CAP, wound healing, inflammation, IL-6, CXCL10, TNF-α

## Abstract

Wound healing is a complex biological process involving cytokines with four phases: Hemostasis, inflammation, proliferation and remodeling. Understanding the molecular mechanism of the inflammation phase could improve wound healing in the clinic as excess inflammation is a critical point for dysregulation of normal wound healing. Capsaicin (CAP), a major component of chili peppers, is known to exhibit anti-inflammatory properties through a range of different pathways, such as the neurogenic inflammation and nociception pathways. To improve the understanding of the relationship between CAP and wound healing, it is crucial to elucidate the CAP-related molecular panel involved in regulating inflammation. Therefore, the present study aimed to analyze the effects of CAP on wound healing using an *in vitro* cell model and an *in vivo* animal model. Cell migration, viability and inflammation were examined using fibroblasts, and wounds were evaluated in mice under CAP treatment. In the present study, it was found that 10 µM CAP increased cell migration and decreased interleukin 6 (IL-6) expression in *in vitro* cell assays. In the *in vivo* animal experiments, the CAP-treated wounds exhibited lower densities of polymorphonuclear neutrophils and monocytes/macrophages, as well as lower IL-6 and C-X-C motif chemokine ligand 10 protein levels. Furthermore, in CAP-treated wounds, CD31-positive capillaries and collagen deposition at the late phase of wound healing were present at higher densities. In summary, an improvement in wound healing by CAP was shown through suppression of the inflammatory response and amelioration of the repair process. These findings suggest that CAP has potential as a natural therapeutic agent for the treatment of wound healing.

## Introduction

Wound healing is a complex biological process that can be divided into four phases: Hemostasis, inflammation, proliferation and remodeling (1). Understanding the molecular mechanism of each phase, particularly the inflammation phase, may aid the development of improved wound healing treatments (2,3). Avoiding excess inflammation is critical to prevent dysregulation of normal wound healing (4).

The first response to a wound is the formation of a fibrin clot that stops blood flow and provides a scaffold for incoming inflammatory cells (5). Subsequently, neutrophils are activated and monocytes are recruited from the vasculature, which releases cytokines and chemokines to initiate the inflammatory phase (6). Inflammatory signals released from the M1 to M2 macrophage conversion promote tissue repair and stimulate the proliferative phase of wound healing (7–9). An appropriate inflammatory response is crucial for acute wound healing (10–12). Chronic wounds that fail to progress through the orderly phases of healing develop when a wound encounters necrotic tissue, pathogen contamination or immune dysfunction (13,14). Preventing the formation of chronic wounds is imperative for wound healing and is best achieved by raising the inflammatory efficiency to shorten the healing period (5). Therefore, it is important to understand and clarify the underlying molecular and cellular mechanisms of wound healing. A reagent that modulates the various phases of wound healing, including one that protects the wound tissue by decreasing prolonged inflammation, and reconstructs damaged tissue may have treatment benefits (15).

Capsaicin (CAP; also known as 8-methyl-N-vanillyl-trans-6-nonenamide) is one of the naturally pungent components of chili peppers, which are varieties of plants from the genus Capsicum (16). CAP is known to affect inflammation through a variety of different pathways, such as the anti-inflammatory response and nociception pathways (17–23), and while CAP may potentially promote wound healing, little is known regarding the mechanism of this process (24). In the present study, the effects of CAP on wound healing were analyzed using both an *in vitro* cell model and an *in vivo* animal model. Briefly, cell migration, viability and reverse transcription-quantitative PCR (RT-qPCR) assays were used in CAP-treated fibroblasts. Animal wounds in mice with CAP treatment were evaluated by next-generation sequencing (NGS), histopathological and immunohistochemical examinations, and Masson's trichrome staining.

## Materials and methods

### Cell culture

The human diploid dermal fibroblast Hs68 cell line (passage 19), which is frequently used in dermal research and has the characteristics of primary dermal fibroblasts (25), was purchased from the Bioresource Collection and Research Center in the Food Industry Research and Development Institute (Hsinchu, Taiwan). Cells were grown in Dulbecco's Minimal Essential Medium (cat. no. 12800017; Gibco; Thermo Fisher Scientific, Inc.) containing 0.1 mM calcium chloride, 1% penicillin/streptomycin/amphotericin (cat. no. 15240062; Gibco; Thermo Fisher Scientific, Inc.) and 10% fetal bovine serum (cat. no. 26140079; Gibco; Thermo Fisher Scientific) at 37°C in a humidified atmosphere of 95% air with 5% CO_2_. Cells were passaged when the culture reached 70–80% confluency.

### Cell migration assay

Cell migration assays were conducted using an Oris cell migration assembly kit (Platypus Technologies, LLC). Briefly, cells were plated at a density of 3×10^4^ cells/well (95–100% confluency) in Oris 96-well plates and cultured overnight. Cells were then serum-starved for a further 24 h. The Oris stoppers were removed after incubation in serum (10%)-containing medium with 10 ng/ml mitomycin C (cat. no. ab120797; Abcam) for 2 h at 37°C to inhibit proliferation (26). According to the reports of other researchers (3,27), the levels of tumor necrosis factor-α (TNF-α) are elevated in the inflammatory phase of wound healing. Therefore, TNF-α, as an inflammation-stimulating agent, was added to simulate an *in vivo* inflammatory environment when wounds occur. The wells were treated with or without TNF-α (10 ng/ml; cat. no. ab259410; Abcam) and different concentrations of CAP (10, 30, and 100 µM; cat. no. M2028; Merck KGaA). Cells were incubated for 24 h at 37°C to allow for cell migration into the detection zone. The migration area was measured at various time points (0 and 24 h) using a light microscope and ImageJ software (version 1.53k; National Institutes of Health) with the MRI Wound Healing Tool plugin. Cell migration is presented as gap closure percentage, which was calculated using the following formula: Gap closure (%)=[(pre-migration) area-(migration) area] ×100/(pre-migration) area (28).

### Cell viability

Cells were plated in 96-well plates at an initial density of 1×10^4^ cells per well and cultured overnight. Following this, different concentrations (10, 30 and 100 µM) of CAP were added and cells were incubated for a further 24 h at 37°C. After incubation, 10 µl labeling reagent, 3-(4,5-dimethylthiazol-2-yl)-2,5-diphenyl-2H-tetrazolium bromide (MTT), was added to give a final concentration of 5 mg/ml in 90 µl culture medium in each well. Cells were incubated for a further 4 h at 37°C with 5% CO_2_. Subsequently, the culture medium was removed and 200 µl dimethyl sulfoxide was added to each well to dissolve the precipitate. Plates were checked for complete solubilization of the purple formazan crystals and the absorbance of the samples was measured using a microplate reader at a wavelength of 570 nm.

### RT-qPCR

Total RNA from Hs68 cells untreated or treated with TNF-α (10 ng/ml) and 10 µM CAP for 24 h was independently extracted using TRIzol^®^ (Invitrogen; Thermo Fisher Scientific, Inc.) according to the manufacturer's protocol. Subsequently, 1 µg total RNA was reverse-transcribed (an initial incubation at 65°C for 5 min, then at 42°C for 60 min and termination at 70°C for 5 min) to single-stranded cDNA using a high-capacity cDNA Reverse Transcription kit (cat. no. K1622; Thermo Fisher Scientific, Inc.). Interleukin 6 (IL-6) qPCR primers (forward, 5′-GATGAGTACAAAAGTCCTGATCCA-3′ and reverse, 5′-CTGCAGCCACTGGTTCTGT-3′) and a TaqMan probe (Universal Probe Library, Human #40; Roche Diagnostics GmbH) were used to quantify IL-6 mRNA levels using the following thermocycler program: 120 sec at 95°C, followed by 60 cycles at 95°C for 5 sec and 60°C for 30 sec. The housekeeping gene, GAPDH (forward primer, 5′-CTCTGCTCCTCCTGTTCGAC-3′ and reverse primer, 5′-ACGACCAAATCCGTTGACTC-3′; Universal Probe Library, Human #60; Roche Diagnostics GmbH), was used as an internal reference. For control purposes, human reference cDNA (cat. no. 636692; Takara Bio USA, Inc.) was used as a positive control to estimate the relative expression levels in the cells. A non-template reaction was used as a negative control. All qPCR reactions were run in a LightCycler 96 (Roche Diagnostics GmbH) and the data were analyzed using the 2^−ΔΔCq^ method (29).

### Animal model and experimental procedures

A total of 40 C57BL/6JNarl male mice (weight, 21–23 g; age, 6 weeks; supplier, National Laboratory Animal Center, Taipei, Taiwan) were used in the present study. All animals were provided with food, water *ad libitum* and housed individually in properly disinfected cages under the following environmental conditions: Humidity, 50±10%; light, 12/12 h light/dark cycle; and temperature, 23±2°C. All procedures involving experimental animals were approved by The Institutional Animal Care and Use Committee at Cathay General Hospital (Taipei, Taiwan; approval no. CGH-IACUC-111-007) and complied with the Guide for the Care and Use of Laboratory Animals (30). Mice were wounded by punch biopsy as following: Two full-thickness circular wounds (6 mm in diameter) on the backs of mice under isoflurane anesthesia (3–4% for induction and 1–3% for maintenance; Terrell liquid for inhalation; Piramal Critical Care, Inc.). The mice were divided into sham (n=13) and experimental (n=18) groups. The wounds of the experimental mice were treated with 200 µl CAP (10 µM) once daily, using Pluronic F127 gel (25% in saline; Merck KGaA) topically. The wounds of the sham mice were only treated with 200 µl Pluronic F127 once daily. To prevent eschar formation on wounds and scratching by the mice, the wounds were covered with a Tegaderm sterile dressing (cat. no. 1624W; 3M). After recovery from the anesthesia, animals were housed individually in properly disinfected cages and maintained on a 12/12-h light/dark cycle. The mice were provided with food and water *ad libitum*. Wounds were imaged on the given days (days 0, 1, 3, 6, 9 and 12) and the size of the wound area was quantified using ImageJ software, version 1.53k (National Institutes of Health). The wound closure area was calculated by Wilson's formula as a percentage of the original area: Wound closure percentage=[(open area at postoperative day 0-open area at postoperative day X)/(wound area at postoperative day 0)] ×100% (31).

At the determined times, 31 mice (4 for sham group and 6 for CAP group at day 3; 6 for sham group and 7 for CAP group at day 6; and 3 for sham group and 5 for CAP group at day 12) were euthanized with CO_2_ at a displacement rate of 30–70% of the chamber volume/min for 2–3 min. A total of 9 mice (3 mice at day 3, 3 mice at day 6 and 3 mice at day 12) served as controls to monitor hygiene, e.g., parasites, viruses and Mycoplasma infections. No mice were sacrificed by euthanasia due to reaching the humane endpoints, including loss of body weight (20% within 3 days) and abnormal coat condition or posture. To verify animal death, respiratory and cardiac arrest was checked and the animals were maintained under CO_2_ for ≥1 min more after respiratory and cardiac arrest. The wounds and surrounding tissues were excised and collected for NGS, histopathological and immunohistochemical examinations, and Masson's trichrome staining.

### Histopathological evaluation of wound healing

All wound specimens were immersion-fixed in 4% buffered paraformaldehyde at room temperature for 24 h, dehydrated in an automatic tissue processor (Shandon Citadel 1000; Thermo Fisher Scientific, Inc.) and embedded in paraffin wax (SAKURA Tissue-Tek; Sakura Finetek). Paraffin serial sections (5-µm thick) were obtained using a rotary microtome (Accu-Cut SRM 200, Sakura Finetek).

Hematoxylin and eosin (H&E) staining was performed with a DRS 2000 Automated Slide Stainer (Sakura Finetek) as following routine protocols: Deparaffinized, rehydrated and stained with hematoxylin solution at room temperature for 5 min, followed by 5 dips in 1% acid ethanol (1% HCl in 70% ethanol). Before mounting the tissue sections on glass slides, sections were rinsed, stained with eosin solution at room temperature for 3 min, dehydrated with graded alcohol and cleared in xylene.

Masson's trichrome staining was manually performed with a commercial kit (cat. no. ab150686; Abcam) to detect collagen fibers. Briefly, sections were deparaffinized in xylene twice for 5 min, rehydrated (100% ethanol twice for 5 min each, 95% ethanol for 5 min, 85% ethanol for 5 min and then 75% ethanol for 5 min), incubated in Bouin's Fluid at 60°C for 60 min and cooled to room temperature for 10 min. All of the following experimental steps were performed at room temperature. The nuclei were stained with Weigert's Iron Hematoxylin for 5 min and all acidophilic tissue elements (such as cytoplasm, muscle and collagen) were stained with Biebrich Scarlet/Acid Fuchsin solution for 15 min. Collagen fibers were further decolored with the depigmenting agent phosphomolybdic acid/phosphotungstic acid for 15 min and counterstained blue in Aniline Blue solution for 10 min. Subsequently, 1% acetic acid was applied to differentiate the sections for 5 min.

All sections were then washed, mounted with an automatic coverslipping machine (Glas-J1; Sakura Finetek) and images acquired using the ZEISS Axio Scan Z1 slide scanner. Two independent pathologists reviewed the images and QuPath (version 0.3.0; http://qupath.github.io) was used to quantify the positive signals (32).

### Immunohistochemical detection of CD31, IL-6, C-X-C motif chemokine ligand 10 (CXCL10) and TNF-α

All immunohistochemical staining was performed using the Ventana Benchmark GX slide stainer (Roche Diagnostics). Briefly, 5-µm thick paraffin-embedded sections were loaded onto the BenchMark carousel in a closed and fixed program: Deparaffinization was performed with EZ Prep solution (cat. no. 950-102; Ventana Medical Systems) at 75°C for 8 min, antigen retrieval with Cell Conditioning 1 solution (cat. no. 950-124; Ventana Medical Systems) at 95°C for 64 min and incubation with the following manually titrated primary antibody at 37°C for 32 min: Anti-CD31 antibody (1:500; cat. no. ab182981; Abcam), anti-IL-6 antibody (1:200; cat. no. ab208113; Abcam), anti-CXCL10 antibody (1:3,000; cat. no. PAA371Mu01; Cloud-Clone Corp.) and anti-TNF-α antibody (1:750; cat. no. GTX15821; GeneTex, Inc.). The slides were then incubated with HQ Universal Linker (cat. no. 253-4580; Ventana Medical Systems) at 37°C for 8 min and HRP Multimer (cat. no. 253-4581; Ventana Medical Systems) at 37°C for 8 min, and visualization by the Optiview DAB IHC detection kit (cat. no. 760-700; Roche Diagnostics) as a chromogenic substrate. All sections were followed by counterstaining with Hematoxylin II at 25°C for 8 min (cat. no. 790-2208; Ventana Medical Systems) and Bluing Reagent at 25°C for 4 min (cat. no. 760-2037; Ventana Medical Systems). All sections were then washed, mounted and scanned to quantify the positive signals, following the protocols stated in the aforementioned histopathological evaluation of wound healing section.

### NGS analysis

Mice were euthanized through CO_2_-induced asphyxiation as aforementioned. Skin tissues from animals were harvested and immediately stored in RNAlater^®^ solution (Ambion; Thermo Fisher Scientific, Inc.) at 4°C. After 24 h, the tissue samples were transferred to −20°C for storage until analysis. Total RNA was extracted using TRIzol (cat. no. 15596026; Theromo Fisher Scientific, Inc.) according to the manufacturer's instructions. Purified RNA was quantified with an optical density of 260 nm using an ND-1000 spectrophotometer (NanoDrop Technologies; Thermo Fisher Scientific, Inc.) and the quality was verified using a Bioanalyzer 2100 (Agilent Technologies, Inc.) and a RNA 6000 LabChip kit (cat. no. 5067-1511; Agilent Technologies, Inc.). All RNA sample preparation procedures were carried out according to the recommended sequencing protocol (Illumina, Inc.). A SureSelect XT HS2 mRNA Library Preparation kit (cat. no. G9995A; Agilent Technologies, Inc.) was used for library construction followed by size selection using AMPure XP beads (cat. no. A63881; Beckman Coulter, Inc.). Sequences were determined using sequencing-by-synthesis technology (cat. no. 20028312; Illumina, Inc). Briefly, the final loading mRNA (200–400 pM; determined by using Agilent TapeStation System; Agilent Technologies, Inc.) was used and sequenced by applying 2×150 base pairs in paired-end runs. Sequencing data (FASTQ reads) were processed using the base-calling program bcl2fastq v2.20 (Illumina, Inc.).

Differential expression analysis was performed using StringTie (StringTir v2.1.4; http://ccb.jhu.edu/software/stringtie) and DEseq (DEseq v1.39.0) or DEseq2 (DEseq2 v1.28.1) with genome bias detection/correction (33). Functional enrichment assays for differentially expressed genes (DEGs) were performed using clusterProfiler v3.6 (https://bioconductor.org/packages/release/bioc/html/clusterProfiler.html) (34). Genes with low expression levels (<0.3 transcripts per million) in either or both the treated and control samples were excluded. Genes with -Log_10_P>1.3 and significant changes in CAP-treated wounds [fold change (FC)>2.0 for upregulated genes or FC<0.5 for downregulated genes] were selected for further bioinformatic analyses. Then, functional annotation enrichment Gene Ontology (GO; http://geneontology.org/) and Kyoto Encyclopedia of Genes and Genomes (KEGG; http://www.genome.jp/kegg/) analyses were conducted using the NGS RNA-sequencing (RNA-seq) data to determine the top significant genes associated with CAP treatment. The Database for Annotation, Visualization, and Integrated Discovery (DAVID; version 6.8; http://david.ncifcrf.gov/summary.jsp) was used to select the enriched DEGs (35), and P<0.05 was considered to indicate a statistically significant difference.

### Statistical analysis

All data, which were repeats of at least three independent experiments, are presented as the mean ± standard deviation. Differences between two groups were compared using an unpaired Student's t-test and three or more groups were compared by one-way ANOVA followed by an appropriate post hoc test, including the Bonferroni test for cell viability and migration, and the least significant difference post hoc test for pairwise comparisons of gene expression. All statistical analyses were performed with SPSS 15.0 software (IBM SPSS Statistics). P<0.05 was considered to indicate a statistically significant difference.

## Results

### Changes in Hs68 cell characteristics in the presence of CAP

**Figure 1.**
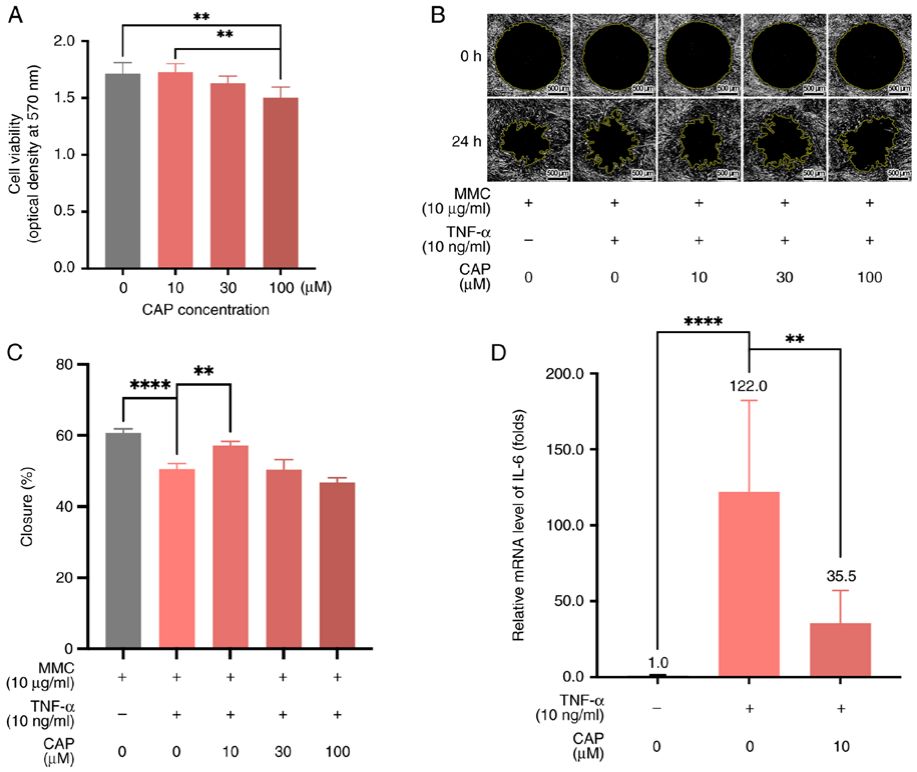
Changes in Hs68 cell characteristics in the presence of CAP. Hs68 cell characteristics were examined following treatment of cells with different CAP concentrations (A) Cell viability, (B) migration (cells were also treated with MMC and TNF-α) and (C) percentage closure based on the cell migration assay were determined. (D) Relative mRNA levels of IL-6 in Hs68 cells treated with TNF-α and CAP. Scale bar, 500 µm. **P<0.01, ****P<0.0001. CAP, capsaicin; IL-6, interleukin 6; MMC, mitomycin C; TNF-α, tumor necrosis factor-α.

When the viability of Hs68 human foreskin fibroblasts treated with different concentrations of CAP (0, 10, 30, 100 µM) were compared, there was a non-significant difference in the viability of cells treated with 10 µM CAP vs. 0 µM CAP (Fig. 1A). However, Hs68 cells exhibited a statistically significant decrease in viability (0 µM CAP vs. 100 µM CAP and 10 µM CAP vs. 100 µM CAP; both P<0.01) upon increasing the concentration of CAP to 100 µM (Fig. 1A). When Hs68 cells were treated with mitomycin C (10 µg/ml) to repress cell proliferation and TNF-α (10 ng/ml) to impair cell migration (36,37), 10 µM CAP effectively restored the migratory ability of Hs68 cells in the cell migration assay (Fig. 1B and C). In addition, 10 µM CAP decreased the mRNA levels of IL-6 in TNF-α-treated Hs68 cells from 122.0-fold to 35.5-fold, relative to the untreated control cells (Fig. 1D). This indicated that 10 µM CAP could change the inflammation status associated with the process of wound healing.

### Improvement of wound healing in vivo by CAP treatment

**Figure 2.**
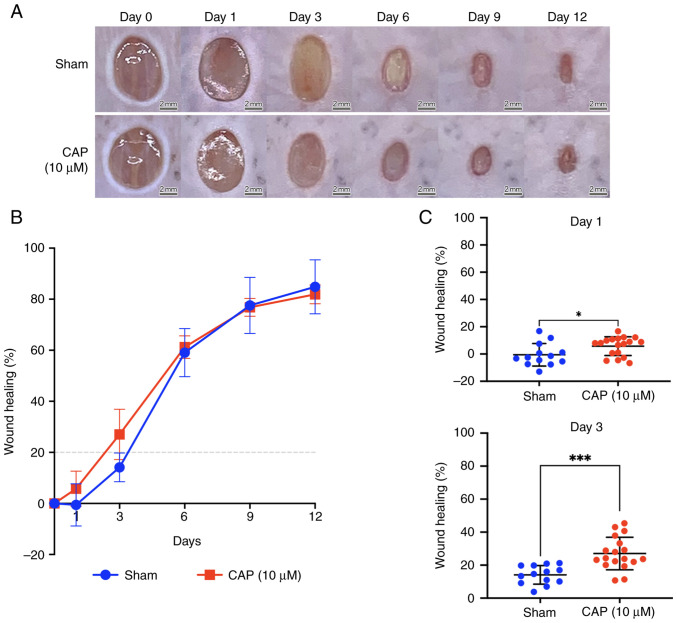
Improvement of wound healing *in vivo* by CAP treatment. (A) Representative images for sham and CAP-treated wounds. Images were captured post-injury at days 0, 1, 3, 6, 9 and 12. Scale bar, 3 mm. (B) Relative percentage of wound healing. Closure areas were determined by ImageJ software and wound healing was calculated relative to the area at day 0. (C) Healing percentage for each wound at days 1 and 3. Sham, n=13 and CAP, n=18. *P<0.05, ***P<0.001 (Student's independent t-test). CAP, capsaicin.

For the wound healing experiments, 6-week-old C57BL/6JNarl mice were selected. A total of two full-thickness circular wounds (6 mm in diameter) were created on the back of mice by punch biopsy on day 0 (Fig. 2A). Briefly, the wound healing was improved by 10 µM CAP treatment compared with the healing in the sham group at days 1 and 3 of wound healing (Fig. 2B). In particular, on days 1 and 3, the wound healing differed significantly between the sham group and 10 µM CAP-treated mice (day 1, P<0.05; day 3, P<0.001; Fig. 2C) while no significant difference of wound healing percentages between these two groups was detected at days 6, 9, and 12.

### Histological difference between wounds with and without CAP treatment

**Figure 3.**
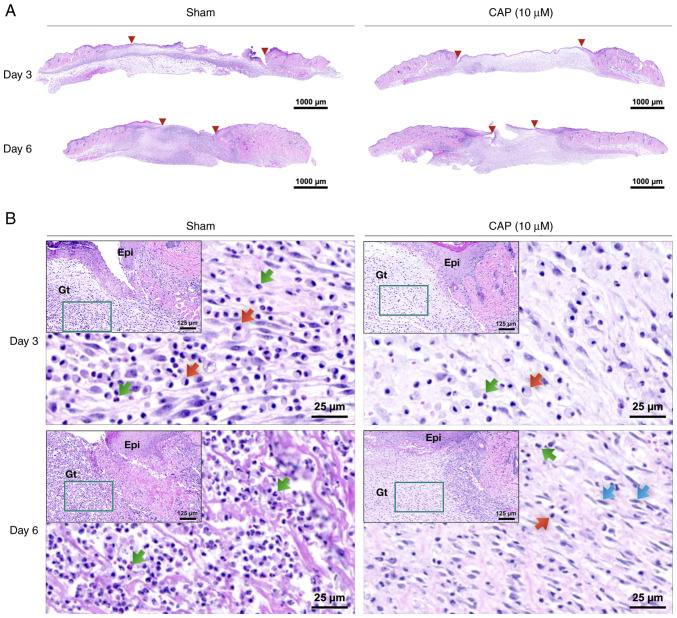
Histological difference between wounds with and without CAP treatment. (A) Sections with hematoxylin and eosin staining of wounds. Red arrowheads indicate the wound region. Scale bar, 1,000 µm. (B) Higher magnification images of the righthand side of the wound edge. Scale bar, 125 µm. The insets (dark green square) indicate the area shown in the highest magnification image (scale bar, 25 µm) at the wound edge. Green arrows indicate polymorphonuclear neutrophils, red arrows indicate monocytes/macrophages and blue arrows indicate fibroblasts. CAP, capsaicin; Epi, epidermis; Gt, granulation tissue.

The width of wounds was measured on post-injured and H&E-stained skin sections (between two red triangles at days 3 and 6 in Fig. 3A, respectively) by QuPath (version 0.3.0). Briefly, H&E staining indicated that the size of the 10 µM CAP-treated wounds was smaller than that of wounds from animals without any CAP treatment (sham group) on days 3 (sham: 5,295±458 µm, and 10 µM CAP: 4,032±275 µm) and 6 (sham: 2,398±296 µm, and 10 µM CAP: 2,276±338 µm). Furthermore, an inflammatory marker, TNF-α, was detected to evaluate the changes of *in vivo* environment caused by CAP treatment. It was found that a weaker signal for TNF-α was detected in the CAP-treated group compared with the sham group (Fig. S1).

In the early phases (days 3 and 6), the 10 µM CAP-treated wounds exhibited lower densities of polymorphonuclear neutrophils (PMNs) and monocytes/macrophages (MMs) compared with wounds of mice in the sham group (Figs. 3B and S2). When compared with the wounds of the sham group, more fibroblasts migrated to the edges of the 10 µM CAP-treated wounds on day 6 (Fig. 3B).

### Blood vessel maturation and collagen deposition in wounds with and without CAP treatment

**Figure 4.**
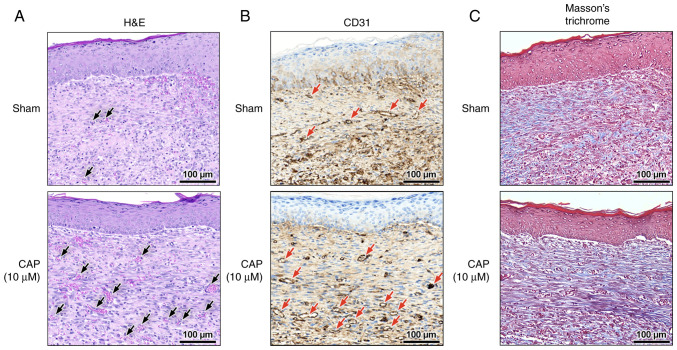
Blood vessel maturation and collagen deposition in wound healing at day 12. (A) Histological sections of blood vessel and (B) immunohistochemical staining of CD31. Red arrows indicate the blood vessels and CD31^+^ endothelial cells, respectively. (C) Collagen alignment indicated by Masson's trichrome stain. Sham wounds were treated with only pluronic F127. Scale bar, 100 µm. CAP, capsaicin; H&E, hematoxylin and eosin.

At the late phase (day 12) of wound healing, blood vessel maturation (Fig. 4A) and CD31^+^ endothelial cells (Figs. 4B and S3A) were frequently encountered in the 10 µM CAP-treated wounds. In addition, in the 10 µM CAP-treated wounds, the remodeling of the granulation layer and the collagen alignment were improved at day 12 (Figs. 4C and S3D, respectively), but not at day 3 (Fig. S3B) and day 6 (Fig. S3C). Notably, all wounds with or without CAP treatment were re-epithelized at day 12 (Fig. 4A).

### CAP-related genes in wound healing based on GO and KEGG pathways

**Figure 5.**
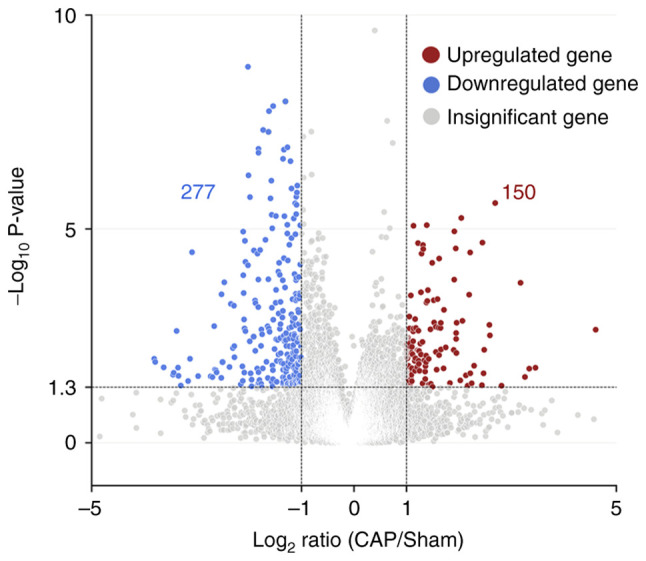
Volcano plot showing the differential pattern of CAP-related genes at day 3. The differentially expressed genes following CAP treatment in wounds at day 3 were explored using next generation RNA-sequencing. Gene annotations of all 427 (150+277) candidates were obtained using the Database for Annotation, Visualization, and Integrated Discovery bioinformatics resources. Red circles indicate upregulated genes with P<0.05 and FC>2.0, blue circles indicate downregulated genes with P<0.05 and FC<0.5, and gray circles indicate genes without significant changes. CAP, capsaicin; FC, fold change.

**Figure 6.**
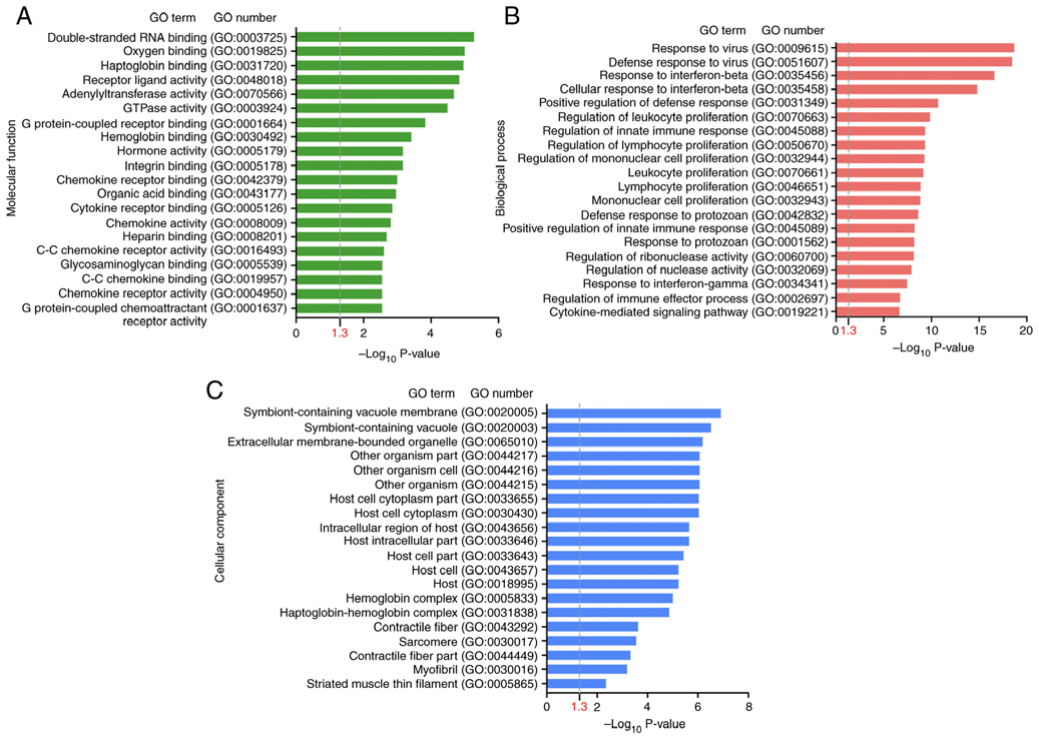
Top 20 GO terms in different categories. (A) Molecular functions, (B) biological processes and (C) cellular components significantly enriched in wounds after 3 days of capsaicin treatment. All GO terms had a P-value <0.05 (−Log_10_P>1.3). GO, Gene Ontology.

To investigate which CAP-induced DEGs were involved in wound healing, the DEGs following CAP treatment in wounds on day 3 were first explored using the NGS RNA-seq data. As shown in Fig. 5, in comparison with wounds without any CAP treatment (sham group), a volcano plot denoted the upregulated (150 genes, log_2_FC ≥1) and downregulated (277 genes, log_2_FC ≤-1) candidates with statistical significance (−log_10_P >1.3) in the wounds with 10 mM CAP treatment for 3 days. On average, ~60% (molecular functions: 60.7%, 259 of 427; biological processes: 59.0%, 252 of 427; cellular components: 61.1%, 261 of 427) of genes were annotated and GO enrichment analysis was performed. The top 20 GO terms in each category (molecular functions, biological processes and cellular components) for pathways enriched in wounds after 3 days of CAP treatment are listed in Fig. 6A-C. The inflammation-related GO terms (Table I) and their prevalent DEGs (Table II) were further selected. For example, IL-6 was in the category of biological processes, interferon-induced proteins (interferon-inducible GTPase 1 and guanylate binding proteins) was in the category of cellular components and CXCL10 was in the category of molecular functions.

**Figure 7.**
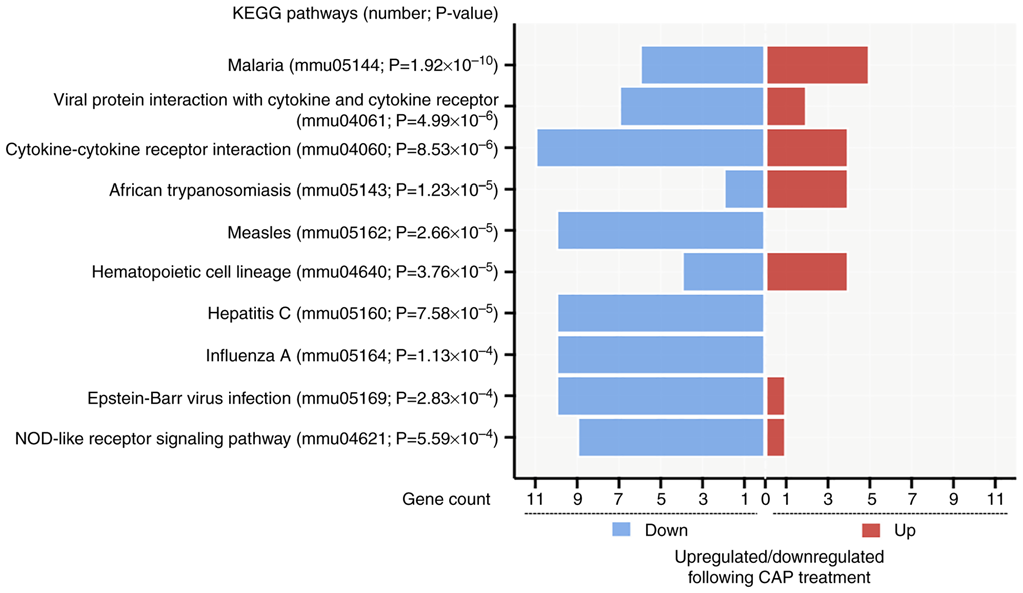
KEGG pathways related to CAP treatment in wound healing. Upregulation/downregulation following CAP treatment is indicated at the bottom of the plot. CAP, capsaicin; Down, downregulated; Up, upregulated; KEGG, Kyoto Encyclopedia of Genes and Genomes; NOD, nucleotide oligomerization domain.

In addition, pathways related to CAP treatment in wound healing were also identified by analyzing KEGG pathways. From the 427 input DEGs, only 100 annotated genes with statistical significance were selected from the top 10 KEGG pathways (P<0.05) (Fig. 7). As depicted in Fig. 7, the pathway of ‘cytokine-cytokine receptor interaction’ (mmu04060; P=8.529×10^−6^) had the most genes, with 11 downregulated genes [CXCL10, CD40, C-X3-C motif chemokine receptor 1, IL-6, bone morphogenetic protein 8a, interleukin 2 receptor subunit α, chemokine (C-C motif) ligand 12, chemokine (C-C motif) receptor 2, C-X-C motif chemokine ligand 9, CD27 and tumor necrosis factor (ligand) superfamily, member 4] and four upregulated genes (GM13306, interleukin 7 receptor, growth differentiation factor 15 and pro-platelet basic protein). Notably, two proinflammatory mediators, CXCL10 and IL-6 (38), present in the GO enrichment analysis were also found in the downregulated gene list of ‘cytokine-cytokine receptor interaction’.

### Immunohistochemical staining of CAP-treated wounds for IL-6 and CXCL10

**Figure 8.**
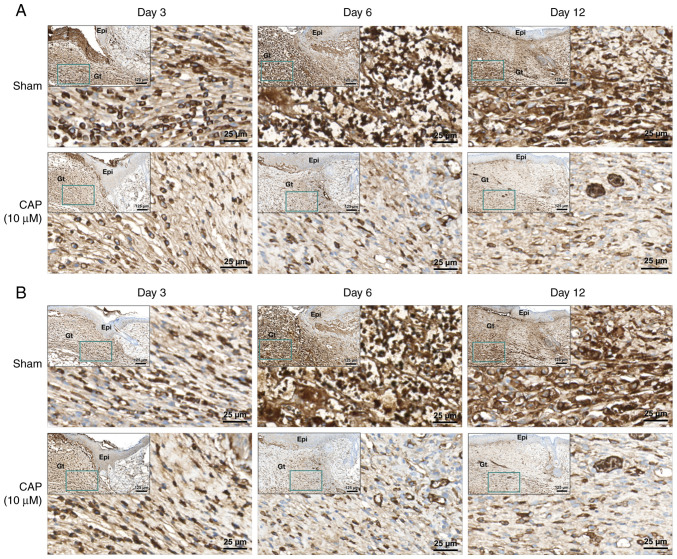
Immunohistochemical staining of CAP-treated wounds. Wound tissues were stained using (A) anti-interleukin 6 and (B) anti-C-X-C motif chemokine ligand 10 antibodies on days 3, 6 and 12 of wound healing. The images at lower magnification (scale bar, 125 µm) are of the righthand side edge of the wound and the insets (dark green square) indicate the location of the highest magnification (scale bar, 25 µm) images in the wound edge. CAP, capsaicin; Epi, epidermis; Gt, granulation tissue.

Upregulation of IL-6 and CXCL10 in wounds increases inflammatory responses and may impair wound healing (39,40). In the present study, the protein levels of IL-6 (Figs. 8A and S4) and CXCL10 (Figs. 8B and S5) were examined in the wound tissues of mice receiving CAP treatment for 3, 6 and 12 days. As shown in Figs. 8, S4A and S5A, higher levels of IL-6 and CXCL10 were detected in granulation tissue of the sham group than those in the CAP-treated group.

## Discussion

The inflammatory response following tissue injury serves important roles in wound healing (10). Different factors or agents that regulate inflammation to affect wound healing have been identified (15,41). Some natural products are known to improve wound healing (42,43). For example, different concentrations of polyherbal extract (*Zingiber officinale, Capsicum annuum, Curcuma longa* and *Cinnamomum verum*) with anti-inflammatory potential to repress the inflammatory cytokines have been characterized (44,45). These observations are consistent with our previous reports demonstrating that topical curcumin could accelerate wound healing by regulating inflammation through an appropriate delivery system (31,46). The present study demonstrated that, in addition to curcumin, CAP also improved wound healing through suppression of the inflammatory response. As reviewed by Habtemariam (47), CAP exerts anti-inflammatory effects by reducing NF-κB phosphorylation to downregulate the levels of IL-6 and TNF-α.

CAP, the most frequently used natural and pungent ingredient of *Capsicum* worldwide, is known to be an agonist of transient receptor potential vanilloid 1 (TRPV1) (48). TRPV1 can be activated by CAP, and subsequently, sodium and calcium ions enter the cell (49). These ion flows depolarize nociceptive neurons to cause action potential discharge and spiciness (49–51), and an analgesic effect due to channel desensitization (52,53). In addition to this analgesic effect, the present study demonstrated that CAP can also affect the migration and viability of human skin fibroblasts.

Currently, CAP is used in concentrations of between 0.025% (0.82 mM) and 8% (262 mM) as a cream or patch for the temporary relief of pain (54). Furthermore, the expression of TRPV1 has been noted in various non-neuronal tissues, including skin cells (e.g., fibroblasts and keratinocytes) (55). This indicates that CAP may have a molecular effect on fibroblasts or keratinocytes. In the present study, some inflammation-related factors (e.g., IL-6 and CXCL10) were found to be differentially expressed in mouse skin wounds treated with 10 µM CAP based on the results of the NGS RNA-seq analyses (GSE220619; GEO database). These molecular differences in inflammation may affect the healing process of skin wound repair (41). In addition, H&E staining in the present study demonstrated that immune cells (PMNs and MMs) were less present in the CAP-treated wounds. These histological results partially agree with the report of Bok *et al* (56), which suggested that CAP might induce transition from the proinflammatory to the anti-inflammatory state (56,57).

The effect of CAP on the skin is controversial. The interaction of CAP and TRPV1 can stimulate inflammatory or anti-inflammatory responses (57–60), and also promote cellular proliferation and migration (61,62). However the concentration of CAP is a crucial factor that CAP may impair (at high doses, >100 µM) or improve (at lower doses, <10 µM) wound healing (63,64), not only in the skin but also in the eyes. Sumioka *et al* (65) reported that 10 µM CAP was able to promote corneal wound healing. These results are consistent with the results of the MTT and cell migration assays with different concentrations of CAP (10–100 µM) in the present study. In the present study, using skin fibroblasts and skin wounds in a mouse model, low-dose CAP (10 µM) increased cell migration and induced an anti-inflammatory response. Notably, the rate-limiting stage of chronic wound healing has been found to be the inflammation phase (66). This suggests that inflammatory responses in wounds could be an important step and related to the future development of wound healing treatments (9). As reported by Xu *et al* (67), shortening prolonged inflammation is effective for increasing the wound healing rate. In the present study, 10 µM CAP had a positive effect on skin wound healing. In addition, the decreased levels of TNF-α, IL-6, and CXCL10 in CAP-treated wounds from the IHC results were associated with the controlled inflammatory responses and these molecular changes may improve wound healing. Taken together, CAP had an anti-inflammatory effect in this context.

In addition to prolonged inflammation, poor angiogenesis is also an important factor that impedes successful wound healing (68). Conversely, any biological, pharmaceutical or cell-based therapies that appropriately increase wound vascularity are beneficial for healing cutaneous wounds (69). More specifically, angiogenic capillaries that form a microvascular network throughout the granulation tissue are necessary to heal wounds (70). This is also consistent with the results of the present study with regard to the increased CD31^+^ endothelial cells, which demonstrated that angiogenic capillaries were present at a higher density in the CAP-treated wounds. Neo-vessel formation may be the result of collagen alignment in the wound area that speeds up the local circulation of essential nutrients, immune cells and oxygen to improve wound healing (71,72).

In the present study, the GO and KEGG pathways included numerous genes involved in virus infection (Table SI). This natural product, CAP, further protects the liver by hindering the entry of hepatitis C virus into hepatocytes by decreasing the expression of the claudin family proteins (73). This may explain why, in the present study, the CAP-treated wound contained downregulated genes related to ‘hepatitis C’ (mmu05160 in KEGG pathway) (74,75). Furthermore, the present results from NGS RNA-seq also indicated that several interferons, inducible large GTPases and guanylate binding proteins (GBPs) were downregulated by CAP in wounds. The decreased GBP3 may be associated with a decrease in interferon-g (76), which has been reported to inhibit the formation of new granulation tissue and wound healing (77–79). Further studies will be required to dissect the effect of CAP, capillary angiogenesis and interferon-γ interactions on wound healing in skin fibroblasts or wounds *in vivo*.

One limitation of the present study is that there is a substantial difference between human and mouse skin (80). The major difference, the loose skin of mice, makes wound healing predominantly occur by contraction, whether the wounds are treated with or without CAP. Instead, the microenvironment of wounds was analyzed in the present study to evaluate the efficacy of CAP treatment. Furthermore, the splinting model may also be used in the future, as it is an easy, standardizable and replicable model for human-like wound healing (81).

The results of the present study may be useful in the design and development of novel processes, technologies or drugs for improved management of wound care. In conclusion, CAP facilitated wound healing by attenuating inflammatory response, increasing angiogenic capillaries and collagen deposition, and decreasing interferon inducible large GTPases, and has a potential application as a therapeutically natural pungent ingredient for wound healing, even for wounds with potentially severe or prolonged inflammation.

## Supplementary Material

Supporting Data

Supporting Data

## Figures and Tables

**Table I.** GO term enrichment analysis of DEGs associated with inflammatory responses in capsaicin-treated wounds.

Category	GO term	GO number	P-value
Biological process	Response to interferon-beta	GO:0035456	2.47×10^−17^
	Cellular response to interferon-beta	GO:0035458	1.58×10^−15^
	Regulation of leukocyte proliferation	GO:0070663	1.39×10^−10^
	Regulation of innate immune response	GO:0045088	4.60×10^−10^
	Regulation of lymphocyte proliferation	GO:0050670	4.65×10^−10^
	Regulation of mononuclear cell proliferation	GO:0032944	5.33×10^−10^
	Leukocyte proliferation	GO:0070661	6.94×10^−10^
	Lymphocyte proliferation	GO:0046651	1.33×10^−9^
	Mononuclear cell proliferation	GO:0032943	1.48×10^−9^
	Positive regulation of innate immune response	GO:0045089	5.71×10^−9^
	Response to interferon-gamma	GO:0034341	3.46×10^−8^
	Regulation of immune effector process	GO:0002697	1.92×10^−7^
	Cytokine-mediated signaling pathway	GO:0019221	2.21×10^−7^
Molecular function	Chemokine receptor binding	GO:0042379	1.01×10^−3^
	Cytokine receptor binding	GO:0005126	1.41×10^−3^
	Chemokine activity	GO:0008009	1.57×10^−3^
	C-C chemokine receptor activity	GO:0016493	2.49×10^−3^
	Chemokine receptor activity	GO:0004950	2.80×10^−3^
	C-C chemokine binding	GO:0019957	2.80×10^−3^
Cellular component	Symbiont-containing vacuole membrane	GO:0020005	1.23×10^−7^
	Symbiont-containing vacuole	GO:0020003	3.04×10^−7^
	Extracellular membrane-bounded organelle	GO:0065010	6.51×10^−7^
	Other organism	GO:0044215	8.55×10^−7^
	Other organism cell	GO:0044216	8.55×10^−7^
	Other organism part	GO:0044217	8.55×10^−7^
	Host cell cytoplasm	GO:0030430	9.14×10^−7^
	Host cell cytoplasm part	GO:0033655	9.14×10^−7^
	Host intracellular part	GO:0033646	2.23×10^−6^
	Intracellular region of host	GO:0043656	2.23×10^−6^
	Host cell part	GO:0033643	3.72×10^−6^
	Host	GO:0018995	5.89×10^−6^
	Host cell	GO:0043657	5.89×10^−6^

GO, Gene Ontology; DEGs, differentially expressed genes.

**Table II.** Enriched genes from the Gene Ontology analysis with differential expression in CAP-treated wounds.

Gene name	NCBI gene ID	Upregulated or downregulated in CAP-treated wounds
GBP3	55932	Downregulated
IIGP1	60440	Downregulated
GBP10	626578	Downregulated
GBP8	76074	Downregulated
GBP9	236573	Downregulated
IL-6	16193	Downregulated
ARG1	11846	Downregulated
TLR9	81897	Downregulated
CCR2	12772	Downregulated
CD40	21939	Downregulated
TNFSF4	22164	Downregulated
IGF2	16002	Downregulated
SLC4A1	20533	Upregulated
GPNMB	93695	Upregulated
CXCL10	15945	Downregulated

ARG1, arginase, liver; CAP, capsaicin; CCR2, chemokine (C-C motif) receptor 2; CXCL10, C-X-C motif chemokine ligand 10; GBP3, guanylate binding protein 3; GBP8, guanylate-binding protein 8; GBP9, guanylate-binding protein 9; GBP10, guanylate-binding protein 10; GPNMB, glycoprotein (transmembrane) nmb; IGF2, insulin-like growth factor 2; IIGP1, interferon inducible GTPase 1; IL-6, interleukin 6; NCBI, National Center for Biotechnology Information; SLC4A1, solute carrier family 4 (anion exchanger), member 1; TLR9, toll-like receptor 9; TNFSF4, tumor necrosis factor (ligand) superfamily, member 4.

## Data Availability

The datasets generated and/or analyzed during the current study are available in the in the NCBI GEO database (https://www.ncbi.nlm.nih.gov/geo/query/acc.cgi?acc=GSE220619) with Accession Number GSE220619.

## Abbreviations

CAP: capsaicin
TNF-α: tumor necrosis factor-α
IL-6: interleukin 6
CXCL10: C-X-C motif chemokine ligand 10
