# Supplementary material for: Improvement of wound healing by capsaicin through suppression of the inflammatory response and amelioration of the repair process
Source: Mol Med Rep. 2023 Jun 30;28(2):155. doi: 10.3892/mmr.2023.13042 (PMC10350740; doi:10.3892/mmr.2023.13042)

Figure S1. Immunohistochemical staining of CAP-treated wounds for TNF-α. Wound tissues were stained using an anti-TNF-α antibody on days 3, 6 and 12. The images at lower magnification (scale bar, 125 μm) are of the righthand side edge of the wound and the insets (dark green square) indicate the location of the highest magnification (scale bar, 25 μm) images in the wound edge. CAP, capsaicin; Epi, epidermis; Gt, granulation tissue; TNF-α, tumor necrosis factor-α.

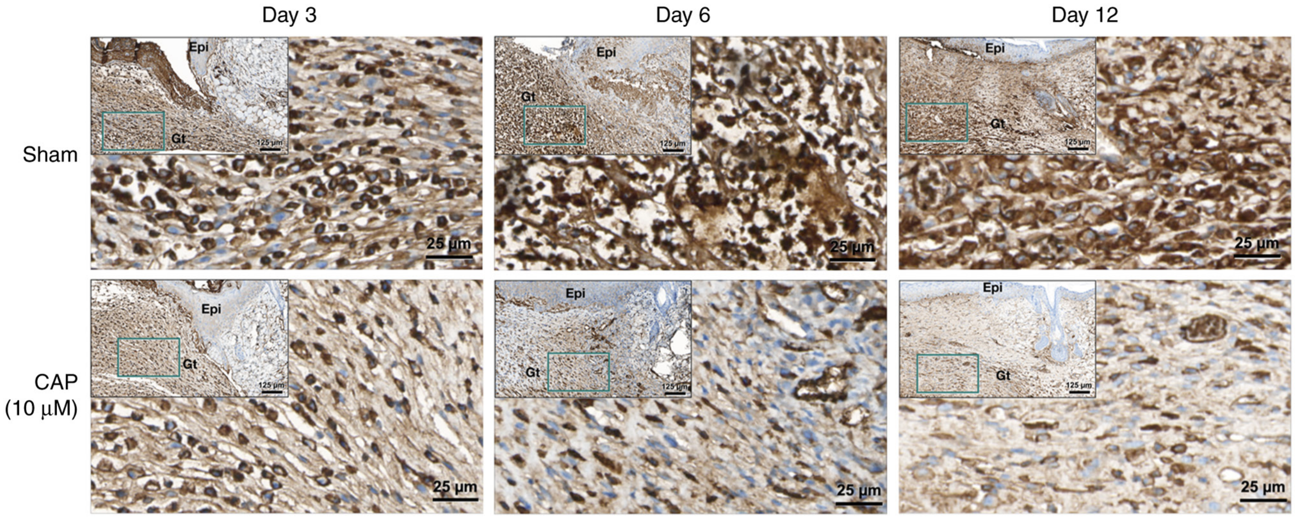

Figure S2. Quantification of the histological difference between wounds with and without CAP treatment. The cell densities of PMNs and MMs per mm^2 were determined on (A) day 3 and (B) day 6. Two independent pathologists reviewed and evaluated the sections of hematoxylin and eosin-stained wounds. *P<0.05; **P<0.01. CAP, capsaicin; MM, monocyte/macrophage; PMN, polymorphonuclear neutrophil.

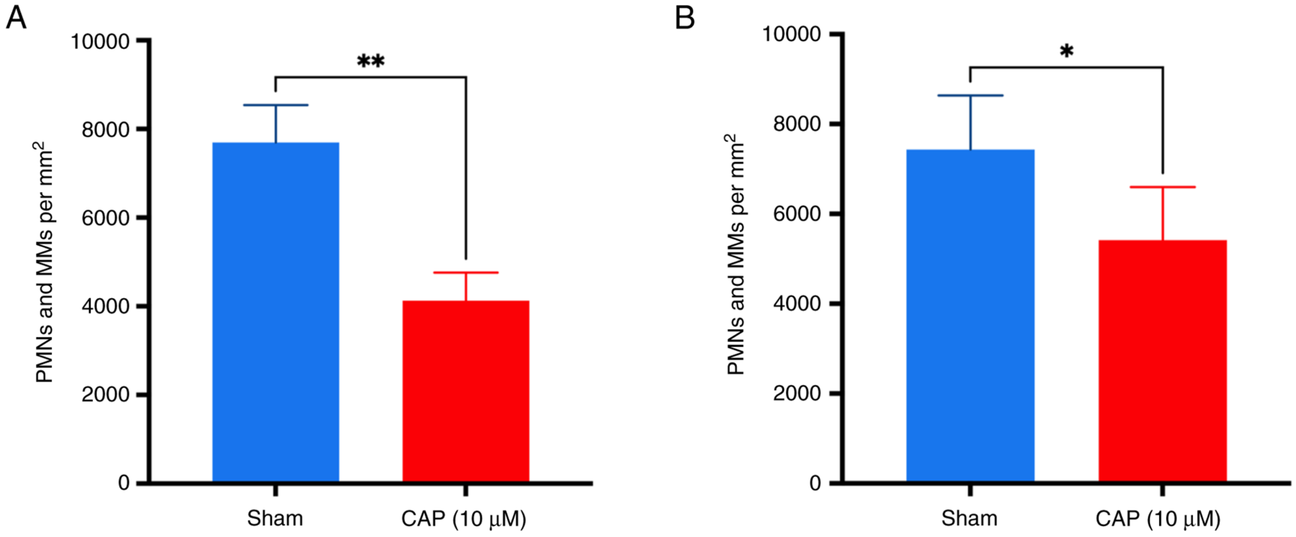

Figure S3. Quantification of the histological difference between wounds with and without CAP treatment. (A) Cell densities of CD31$^+$ cells per mm^2, and percentages of collagen deposition on (B) day 3, (C) day 6 and (D) day 12 were determined. Representative images of collagen deposition are provided alongside the bar charts. The images for day 12 are the same images shown in Fig. 4C but at a higher magnification. Scale bar, 50 μm. Two independent pathologists reviewed and evaluated the sections with immunohistochemical staining and the Masson's trichrome-stained wounds. *P<0.05; ***P<0.001. CAP, capsaicin.

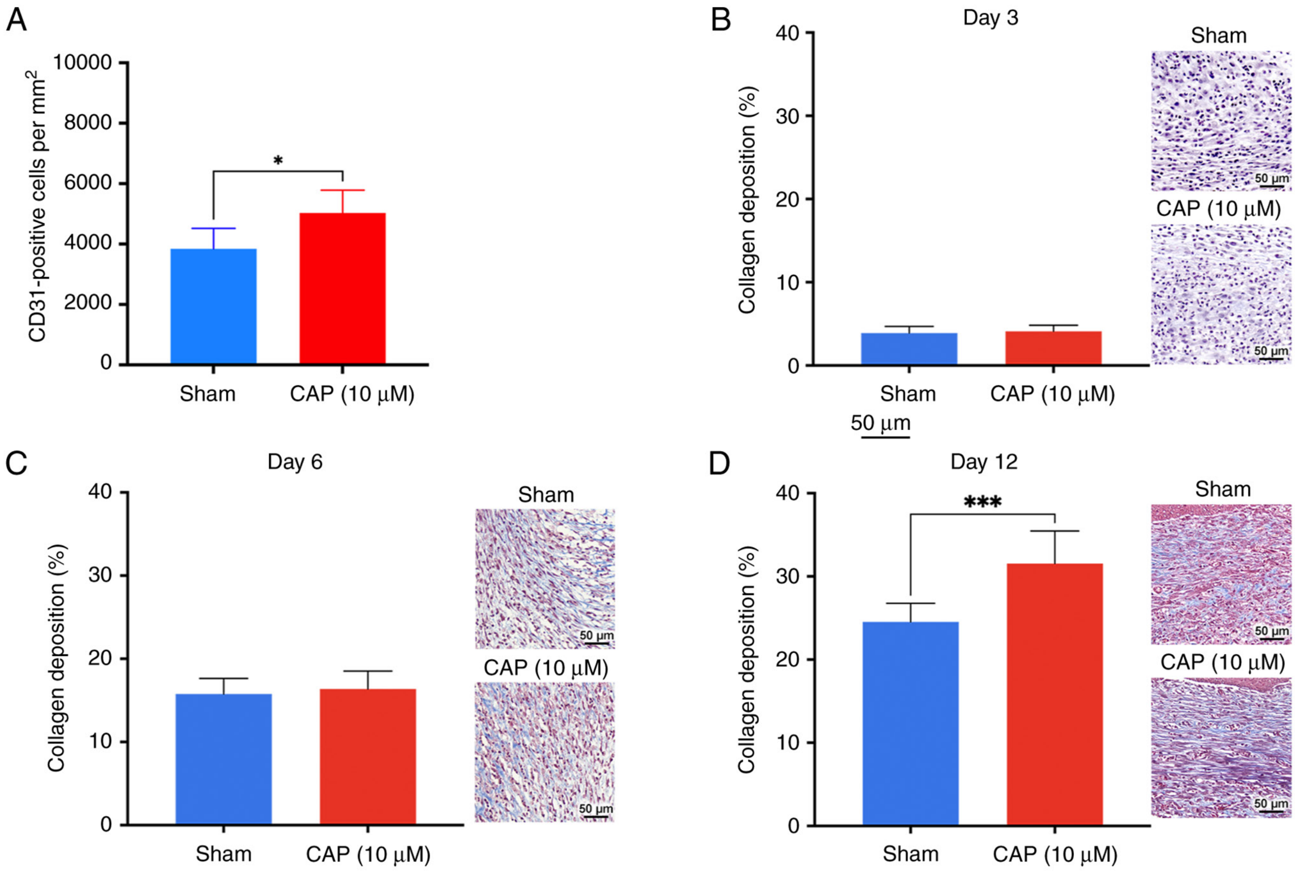

Figure S4. Quantification of the immunohistochemical staining in wounds with and without CAP treatment. The cell densities of IL-6$^+$ cells per mm^2 were determined on (A) day 3, (B) day 6 and (C) day 12. Two independent pathologists reviewed and evaluated the wound sections with immunohistochemical staining. *P<0.05; **P<0.01. CAP, capsaicin; IL-6, interleukin 6.

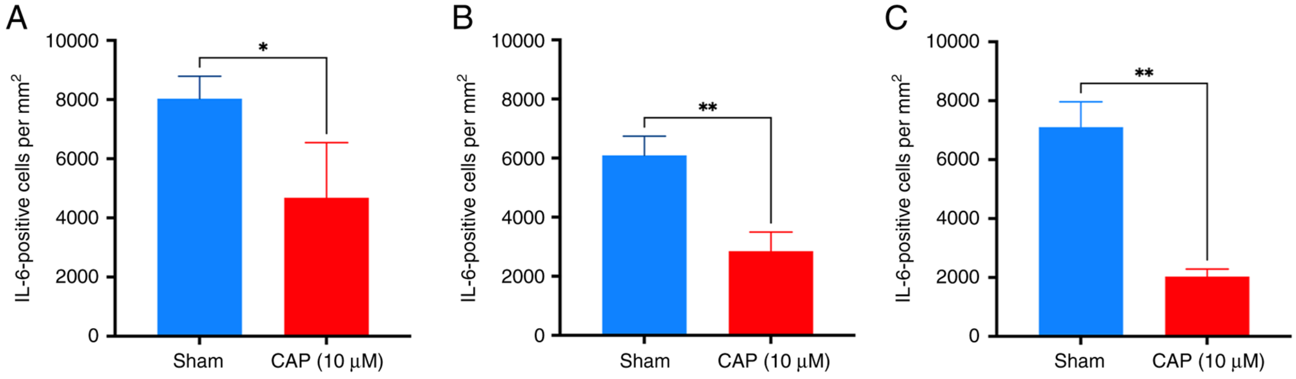

Figure S5. Quantification of the immunohistochemical staining of wounds with and without CAP treatment. The cell densities of CXCL10$^+$ cells per mm^2 were determined on (A) day 3, (B) day 6 and (C) day 12. Two independent pathologists reviewed and evaluated the wound sections with immunohistochemical staining. $^*P<0.05$; $^{**}P<0.01$. CAP, capsaicin; CXCL10, C-X-C motif chemokine ligand 10.

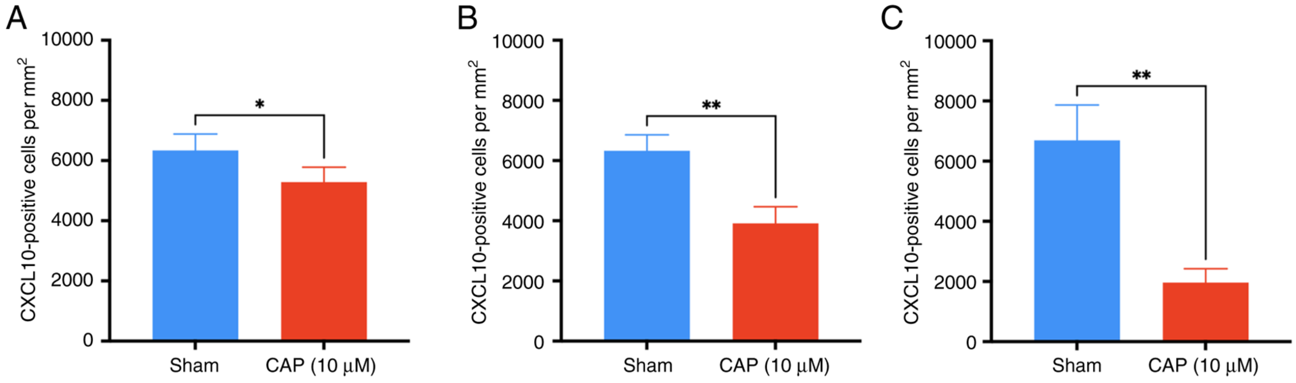

Supplement: Supporting Data [file Supplementary_Data1.pdf]
