# Supplementary material for: Improvement of wound healing by capsaicin through suppression of the inflammatory response and amelioration of the repair process
Source: Mol Med Rep. 2023 Jun 30;28(2):155. doi: 10.3892/mmr.2023.13042 (PMC10350740; doi:10.3892/mmr.2023.13042)
Supplement: Supporting Data [file Supplementary_Data2.pdf]

Table SI. Top 10 significant terms with regard to virus infection in GO and KEGG pathways.

Category	Term	Number	P-value
GO - biological process	Response to virus	GO:0009615	$2.03x10^{-19}$
	Defense response to virus	GO:0051607	$3.42x10^{-19}$
GO - molecular function	Double-stranded RNA binding	GO:0003725	$5.30x10^{-6}$
	Oxygen binding	GO:0019825	$1.00x10^{-5}$
	GTPase activity	GO:0003924	$3.24x10^{-5}$
KEGG	Viral protein interaction with cytokine and cytokine receptor	mmu04061	$4.99x10^{-6}$
	Measles	mmu05162	$2.66x10^{-5}$
	Hepatitis C	mmu05160	$7.58x10^{-5}$
	Influenza A	mmu05164	$1.13x10^{-4}$
	Epstein-Barr virus infection	mmu05169	$2.83x10^{-4}$

GO, Gene Ontology; KEGG, Kyoto Encyclopedia of Genes and Genomes.
